# Glomerular ultrastructural change and vascular endothelial growth factor‐A expression in diabetic cats

**DOI:** 10.1111/jsap.13884

**Published:** 2025-06-04

**Authors:** H. Reyes‐Hughes, E. Bird, C. Neal, R. Noiva, R. R. Foster, S. Satchell, N. Finch

**Affiliations:** ^1^ Langford Vets, Langford House Langford UK; ^2^ Bristol Veterinary School University of Bristol Langford UK; ^3^ Bristol Renal, Bristol Medical School University of Bristol Bristol UK; ^4^ Centre for Interdisciplinary Research in Animal Health, Faculty of Veterinary Medicine University of Lisbon Lisbon Portugal; ^5^ Associate Laboratory for Animal and Veterinary Sciences Lisbon Portugal; ^6^ Present address: The George Veterinary Group Malmesbury UK

## Abstract

**Objectives:**

To evaluate the glomerular ultrastructural changes associated with diabetic nephropathy and quantify glomerular vascular endothelial growth factor‐A expression in cats with diabetes mellitus compared to controls.

**Materials and Methods:**

Transmission electron microscopy was performed to evaluate glomerular ultrastructural changes, including glomerular endothelial cell fenestration density and width, glomerular basement membrane width, podocyte foot process width, and podocyte slit width and density in four diabetic and five control cats. Glomerular vascular endothelial growth factor‐A expression was quantified using immunohistochemistry in eight diabetic and five control cats.

**Results:**

Glomerular ultrastructural change consistent with diabetic nephropathy, including loss of glomerular endothelial cell fenestrations and thickened glomerular basement membrane, was present in diabetic cats. Furthermore, diabetic cats had increased glomerular vascular endothelial growth factor‐A expression.

**Clinical Significance:**

This study demonstrates that diabetic nephropathy may develop in diabetic cats, which although representing early subclinical disease, may contribute to decreased glomerular filtering capacity and proteinuria and therefore requires clinical monitoring.

## INTRODUCTION

Diabetes mellitus (DM) is a common endocrine disease affecting cats with a reported prevalence of 0.58% (O'Neill et al., 2016). It results from loss of insulin‐producing pancreatic beta cells (type 1 DM) and/or peripheral insulin resistance (type 2 DM) (Bloom & Rand, 2013) with type 2 DM considered more common than type 1 in cats (Nelson & Reusch, 2014). Diabetic kidney disease, also known as diabetic nephropathy (DN), is the leading cause of kidney failure in the western world (Duran‐Salgado, 2014). In people, it is identified in both type 1 and 2 diabetes, with 25% to 40% of diabetic people developing DN within 10 to 20 years of diagnosis of DM (Bloom & Rand, 2013). DN is classified into five stages in diabetic people (Haneda et al., 2015): (1) glomerular hyperfiltration and hypertrophy; (2) structural changes to the glomerulus; (3) persistent microalbuminuria; (4) clinical nephropathy with macroalbuminuria, decreased glomerular filtration rate (GFR) and hypertension and (5) end‐stage kidney disease. The first two stages, characterised by glomerular hyperfiltration, hypertrophy and glomerular structural changes, are subclinical and related to microvascular changes in the renal glomeruli. The hallmark of early clinical DN is microalbuminuria (Venkat, 2004). This can progress to overt proteinuria and eventually end‐stage renal disease (Shahbazian & Rezaii, 2013). In the first two early subclinical stages, the changes within the kidney develop at an ultrastructural level and involve the glomerulus. These glomerular ultrastructural changes include glomerular basement membrane (GBM) thickening, loss of glomerular endothelial cell (GenC) fenestrations, podocyte foot process effacement, podocyte slit loss and widening, and mesangial matrix expansion (Osterby, 1993; Satchell, 2012). Visualisation of these ultrastructural changes requires the use of electron microscopy, since light microscopy does not provide the necessary magnification and resolution.

To date, there have been few studies exploring the development of DN in cats. Light microscopy has been used to evaluate the structural changes in the kidneys of cats with and without DM. Whilst changes consistent with DN, including GBM thickening and mesangial matrix increase, were observed in some cats with DM, these were not significantly different from control cats (Zini et al., 2014). An earlier study identified changes consistent with DN in 50% of diabetic cats (*n* = 6) at post‐mortem (Nakayama et al., 1990); however, a control population was not included. A literature search confirmed that there are no studies evaluating the ultrastructural changes in the glomeruli of diabetic cats. The renal functional changes in cats with DM have been reported. Results of studies are inconsistent; however, the high prevalence of overt proteinuria (Al‐Ghazlat et al., 2011) and microalbuminuria (Al‐Ghazlat et al., 2011) may suggest the presence of glomerular filtration barrier damage in diabetic cats. Whilst DM (Greene et al., 2014) and obesity (Perez‐Lopez et al., 2020) have not been reported to be a risk factor for chronic kidney disease (CKD) in cats, this would represent clinical disease. The early subclinical findings associated with DN have not been examined in cats.

The development of DN is complex with many different factors involved. One factor considered to have a key role is vascular endothelial growth factor‐A (VEGF‐A), an angiogenic and endothelial cell growth factor. Within the glomerulus, VEGF‐A is produced by the podocytes and binds to VEGF receptor 2 on the GEnC. VEGF‐A expression has been shown to be increased in both diabetic mice and humans in the early stages of DN before and then decreasing in the later stages (Shibuya, 2011). Glomerular VEGF‐A expression has not been reported in diabetic cats.

Clinically overt DN is not generally recognised in cats, perhaps because they do not survive for a sufficient period of time to develop it or perhaps because they demonstrate true resistance to disease. Yet, studies to evaluate the early glomerular ultrastructural changes that characterise DN to fully understand these postulations have not been undertaken. A literature search of the database Medline (PubMed) was performed with the following keywords: diabetic nephropathy, kidney, cats on February 27, 2025. No other reports of the glomerular ultrastructural changes in cats with diabetes have been found in this search. The study aims were, firstly, to characterise the glomerular ultrastructural changes and, secondarily, to quantify glomerular VEGF‐A expression in cats with DM compared to controls.

## MATERIALS AND METHODS

### Study population and inclusion criteria

Cats included in this study were selected retrospectively from available pathology samples at the Veterinary Pathology Department, Bristol Veterinary School, University of Bristol. Informed consent was gained from owners of cats undergoing post‐mortem examination for retention of tissue for research studies. The study was conducted with approval from the University of Bristol Animal Welfare and Ethical Review Body (UB/17/19). All patients had presented as clinical cases to Langford Vets Small Animal Referral Hospital, Bristol Veterinary School.

Kidney tissue samples from client‐owned cats were collected at post‐mortem within a maximum of 12 hours following euthanasia. A clinical diagnosis of diabetes, defined by a serially increased blood glucose concentration and glucosuria or, where available, increased fructosamine concentration, was confirmed prior to death. The control population consisted of cats in which the disease status did not result in any changes to glucose homeostasis or renal pathology and which had not received any medications affecting glucose homeostasis or renal function.

### Transmission electron microscopy to evaluate glomerular ultrastructural changes

Kidney tissue samples fixed in 10% neutral buffered formalin were further fixed in glutaraldehyde, post‐fixed in 1% osmium tetroxide and *en bloc* stained in uranyl acetate and lead aspartate, followed by ethanol dehydration and embedding in Durcupan resin (Agar Scientific). Sections were cut at 70 to 90 nm using an ultramicrotome. Electron micrographs were acquired using a Technai 12 electron microscope (Thermo Scientific, UK). Standardised image acquisition was performed by acquiring images of two to three glomeruli per cat with six random images per glomerulus (3 from the 12 o'clock position from a low power of the circular glomerulus and 3 from the 6 o'clock, or 9 and 3 o'clock if the other positions were obscured by a urinary or vascular pole). [Correction added on 4 September 2025, after first online publication: The previous sentence has been revised for clarity, changing “two or three glomeruli cat” to “two to three glomeruli per cat” in this version.]

**FIG 1:**
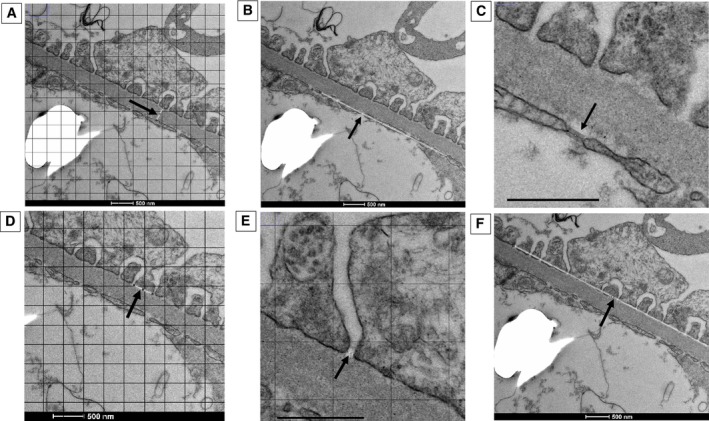
Glomerular ultrastructural measurements taken from transmission electron micrographs. Lines and arrows indicate examples of where measurements were made: (A) glomerular basement width (GBM) measured at randomly determined grid intersections from the glomerular endothelial cell membrane to the podocyte foot process membrane; (B) glomerular endothelial cell (GEnC) fenestration density measured by counting the number of fenestrations per unit length of cytoplasm; (C) GEnC fenestration width measured from the narrowest diameter between two opposing cell membranes, scale bar 500 nm; (D) podocyte foot process width measured at randomly determined grid intersections along the length of the GBM it contacted; (E) podocyte slit width measured at randomly determined grid intersections at the narrowest diameter between two adjacent foot processes, scale bar 500 nm and (F) podocyte slit density measured by counting the number of slit processes per unit length.

Glomerular ultrastructural changes were evaluated by a single investigator masked to the clinical data. Transmission electron micrographs (TEMs) were analysed using image processing software (FIJI) and the following measurements recorded:

- GBM widths were randomly determined by overlaying a grid (area per point: 1 mm^2^) onto each image and measuring the GBM width at the grid intersection. The width was measured from the GenC membrane to the podocyte foot process membrane (phospholipid bilayer) subject to there being two entire cell membranes. The measurement was taken by the shortest route, perpendicular to the GBM edge (Fig 1A).
- GEnC fenestration density and width were measured where GEnC boundaries could be clearly visualised. GEnC fenestration density was determined by counting the number of fenestrations per unit length of the GenC peripheral cytoplasm (Fig 1B). GEnC fenestration width was determined by measuring the diameter of the fenestration at the narrowest distance between the opposing cell membranes (Fig 1C). The GenC fenestration surface area was determined by the percentage of measured GEnC surface area covered by total fenestration widths.
- Podocyte measurements were randomised using a grid overlay as described above. Measurement of podocyte foot process width (Fig 1D) and podocyte slit width (Fig 1E) was performed where the grid intersected over an entire podocyte foot process or slit process. Podocyte foot process width was measured where it contacted the glomerular basement membrane. Podocyte slit width was measured at the narrowest diameter between two adjacent foot processes. Podocyte slit density was determined from the number of podocyte slits covering a length of GBM (Fig 1F).

### Histopathological examination of kidney tissue

Samples of kidney tissue fixed in 10% neutral buffered formalin in which glomerular ultrastructural measurements had been performed were routinely processed and paraffin embedded. Four micrometre sections were stained with haematoxylin and eosin stain for morphological analysis under optical microscopy. A board‐certified veterinary pathologist who was blinded to the disease status of the cats evaluated the kidney sections using the previously published scoring schematic for dichotomous and semiquantitative histologic variables (McLeland et al., 2015). Samples were evaluated by quantifying the % normal parenchyma, tubule degeneration, interstitial inflammation, % cortical scarring and % medullary scarring. These variables were then scored semi‐quantitatively on a scale from either 0 to 3 or 0 to 4. The scoring system is included in Supplementary Material 1.

### Glomerular VEGF‐A expression

Formalin‐fixed, paraffin‐embedded sections from cats in which glomerular ultrastructural measurements had been performed were evaluated for glomerular VEGF‐A expression using immunohistochemistry. In addition, previously processed sections from four additional diabetic cats were available in the University of Bristol pathology archives and included in the study. Sections (4 μm) underwent deparaffinization and hydration steps involving incubation in Histo‐Clear II and decreasing concentrations of ethanol. Antigen retrieval was performed by heating sections in 10 mM sodium citrate buffer (pH 6). Non‐specific IgG binding was blocked with 1% bovine serum albumin (BSA) and 10% normal goat serum in Tris‐buffered saline‐Triton‐X‐100 (0.1%). Sections were incubated in primary antibody sc‐65617 (Santa Cruz Biotechnology, Texas, USA) diluted 1:100 or an IgG control overnight at 4°C. The following day, endogenous peroxidase activity was blocked with 3% (wt/vol) hydrogen peroxide and sections incubated with a horseradish peroxidase‐conjugated secondary antibody specific for the antibody (SignalStain Boost IHC Detection Reagent, Cell Signaling, Danvers, MA, USA). Sections were subsequently incubated with 3,3'‐diaminobenzidine (DAB) substrate (SignalStain DAB substrate, Cell Signaling, Danvers, MA, USA) and counter stained with haematoxylin. Image analysis was performed using an image software program (FIJI) by a blinded investigator. Corrected total staining intensity was determined in three glomeruli per cat and the mean for each cat was used in statistical analyses. Images were obtained at ×40 magnification using light microscopy.

### Statistical analysis

As group sizes were small, the data were analysed using non‐parametric statistical testing. Data between the diabetic and control groups were compared using the Mann–Whitney *U*‐test. Significance was set at P < 0.05.

## RESULTS

### Study population

A total of four diabetic and five control cats were included in the electron microscopy study. Diabetic cats included two male neutered domestic short hairs (DSH), one female neutered DSH and one female neutered Burmese. Two of the diabetic cats were in diabetic ketoacidosis at the time of death, and these cats had a short survival period post‐diagnosis of DM (4 weeks to 2 months). One cat was diagnosed with DM 5 days prior to death and was not in diabetic ketoacidosis, and one cat had a diagnosis of DM for 2 years prior to euthanasia. In the diabetic group, other co‐morbidities were noted at pre‐ and post‐mortem examination. The co‐morbidities were suspected acromegaly, hepatic lipidosis, controlled hyperthyroidism, suspected cholangitis, unknown pancreatopathy (previous pancreatitis or age‐related pancreatic nodules), aortic thromboembolism, hypertrophic cardiomyopathy and Heinz body haemolytic anaemia.

The control population included five cats (three DSH, one Havana Brown and one Maine Coon). There were two male entire, one male neutered and two female neutered cats. Each individual had multiple co‐morbidities noted at post‐mortem examination. The co‐morbidities noted included meningitis, neutrophilic pleural effusion and a mild pericardial effusion, nasal lymphoma, cerebellar eosinophilic encephalitis with mixed inflammatory meningitis and hepatic haemangiosarcoma.

The clinical data from control and diabetic cats are presented in Table 1. Diabetic cats were significantly older than control cats [median (range) 10 (9 to 13) years vs. 5 (1 to 16) years, P = 0.021]. In all cats, multiple measurements of blood glucose were obtained, and the blood glucose measurement at initial presentation was presented. Serum fructosamine was measured in only one of the diabetic cats and was above the normal reference interval at 527 μmol/L. Additionally four diabetic cats were included in the study to evaluate glomerular VEGF‐A expression using immunohistochemistry. The median (range) age of these cats was 11.5 years (9 months to 12 years). Two cats were FN Siamese, one cat was an MN Siamese, and one cat was an FN Burmese. At post‐mortem, it was reported that one cat had a jejunal mass and one cat had an adrenal adenoma. Clinical notes describing the onset of diabetes and urine protein:creatinine ratio (UPC) were not available. Blood glucose was documented in the post‐mortem report from one cat and was 25.6 mmoL/L.

**Table 1:** Clinical data from control and diabetic cats included in the electron microscopy analysis

Median (range)	Control cats (*n* = 5)	Diabetic cats (*n* = 4)	P value
Age (years)	5 (1 to 16)	10 (9 to 13)	0.021
Creatinine (μmol/L)	66 (65 to 117)	99 (31 to 188)	0.700
Urine protein: creatinine ratio	NA	2.23 (0.85 to 4.13)	NA
Systolic blood pressure (mmHg)	149 (120 to 158)	156 (154 to 160)	0.212
Blood glucose (mmol/L)	5.95 (4.50 to 9.10)	17.00 (14.10 to 23.3)	0.020

NA Not available

### Transmission electron microscopy to evaluate glomerular ultrastructural changes

**FIG 2:**
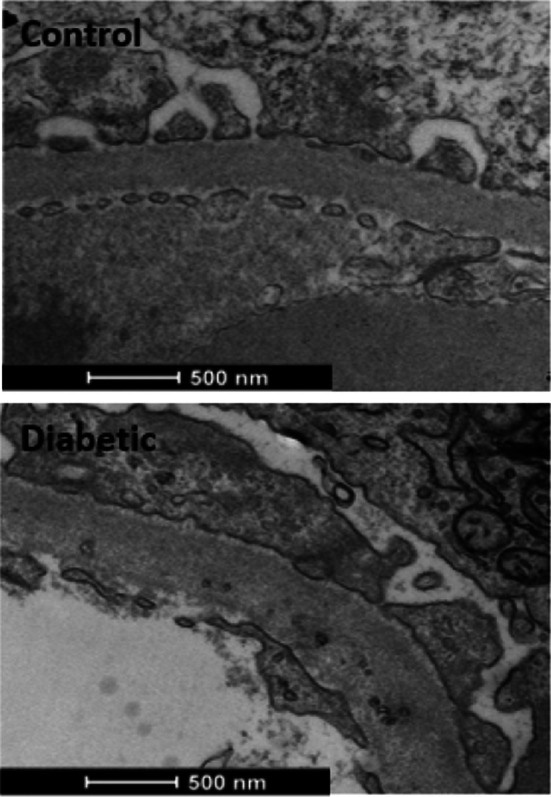
Representative transmission electron micrograph images from control and diabetic cats demonstrating loss of glomerular endothelial cell fenestrations and thickened glomerular basement membrane.

**FIG 3:**
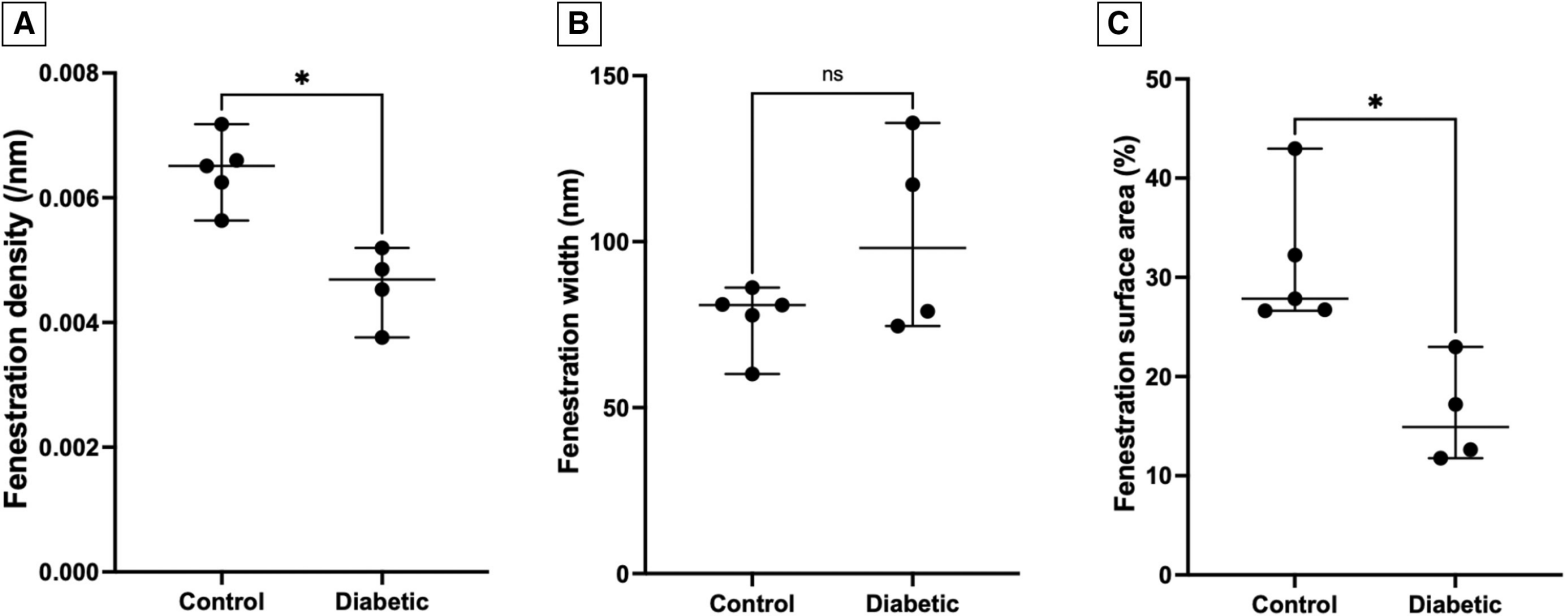
Glomerular endothelial cell fenestration measurements in diabetic and control cats: (A) glomerular endothelial cell fenestration density, (B) glomerular endothelial cell fenestration width and (C) glomerular endothelial cell fenestration surface area. *P < 0.05. ns Not significant.

**FIG 4:**
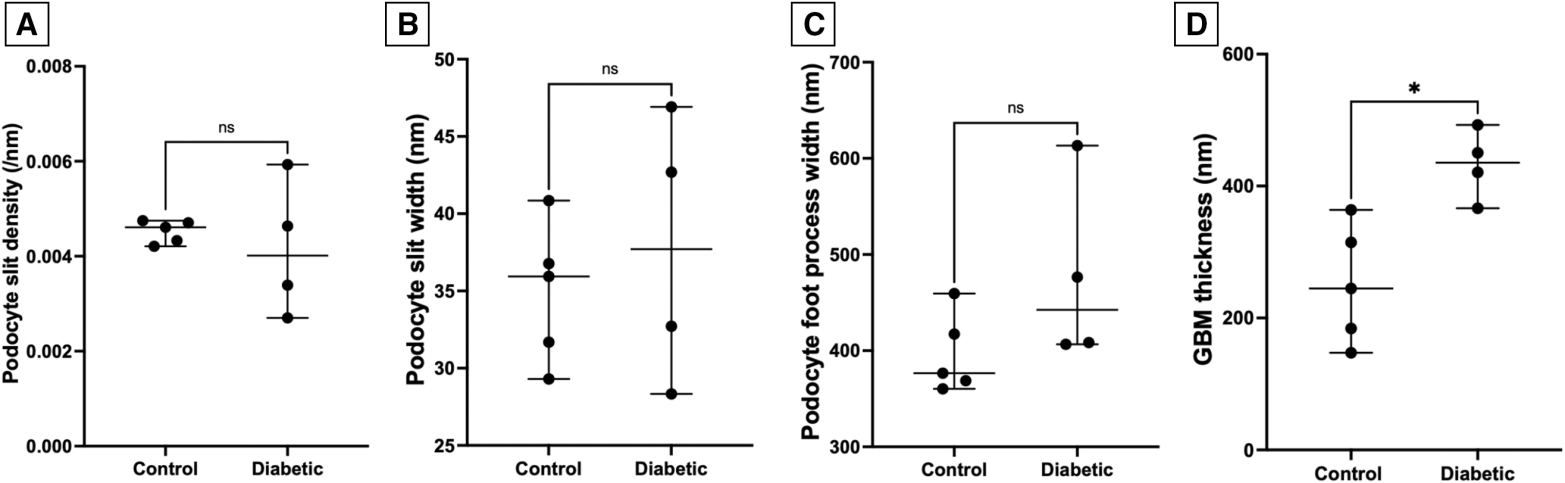
Podocyte and glomerular basement membrane (GBM) measurements in diabetic and control cats: (A) podocyte slit density, (B) podocyte slit width, (C) podocyte foot process width and (D) GBM thickness. *P < 0.05. ns Not significant.

Representative TEM images from control and diabetic cats are presented in Fig 2. The GenC fenestration measurements are presented in Fig 3. GenC fenestration density (Fig 3A) and fenestration surface area (Fig 3C) were significantly decreased in diabetic compared to control cats, whilst fenestration width (Fig 3B) was not significantly different. Podocyte measurements were not significantly different in diabetic compared to control cats (Fig 4A–C); however, GBM width was significantly increased in diabetic compared to control cats (Fig 4D).

### Histopathological examination of kidney tissue

Histopathological scores of kidney tissue from the four diabetic and five control cats in which ultrastructural measurements were performed were not significantly different (Table 2).

**Table 2:** Histopathological scoring of kidney tissue from control and diabetic cats included in the electron microscopy analysis

Variable	Control cats (*n* = 5)	Diabetic cats (*n* = 4)	P value
% normal parenchyma score Median (range)	4 (2 to 4)	4 (1 to 4)	0.732
Tubular degeneration score Median (range)	1 (0 to 1)	2 (2 to 3)	0.143
% interstitial inflammation score Median (range)	1 (0 to 1)	1 (1 to 2)	0.536
% cortical scarring Median (range)	0 (0 to 1)	0 (0 to 1)	0.999
% medullary scoring Median (range)	1 (0 to 3)	1 (0 to 4)	0.999

### Glomerular VEGF‐A expression

**FIG 5:**
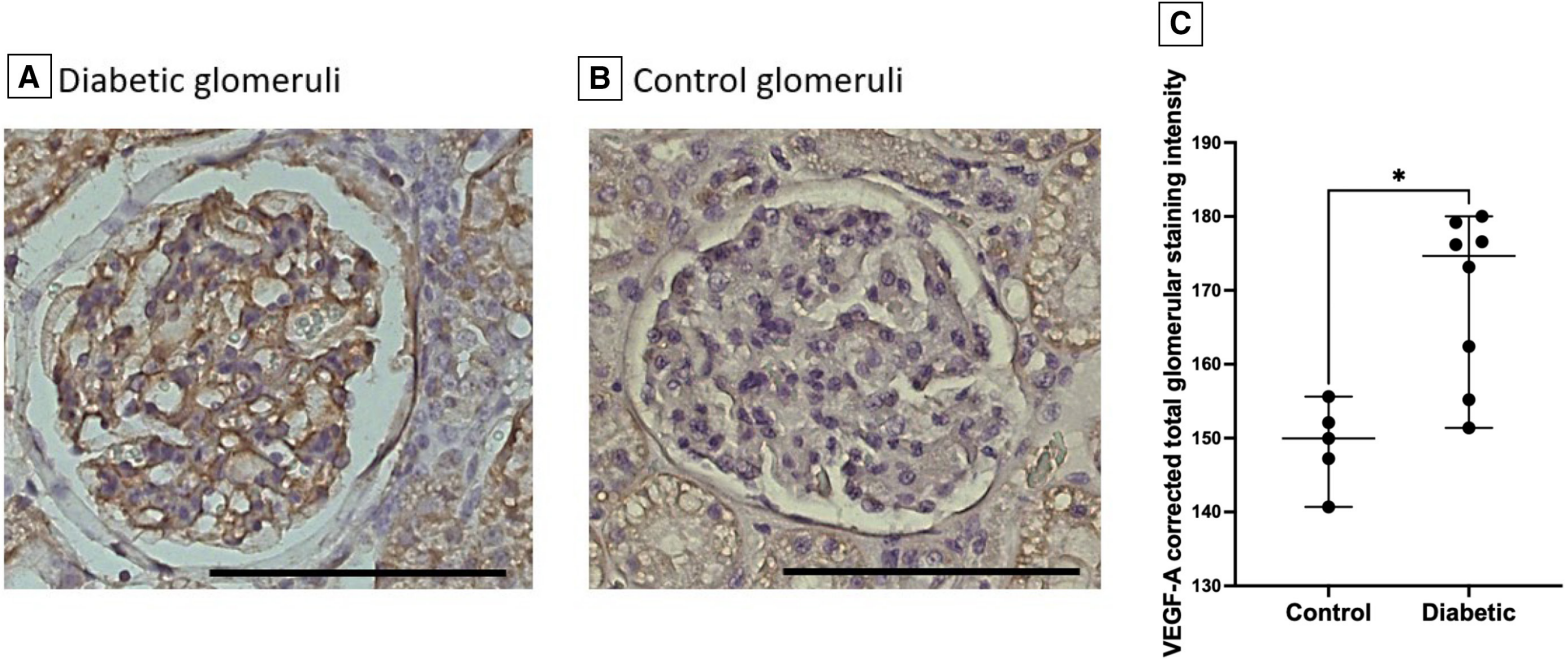
Glomerular vascular endothelial growth factor‐A (VEGF‐A) expression in control and diabetic cats: (A) representative image of glomerular VEGF‐A expression in a diabetic cat glomerulus, scale bar 100 μm; (B) representative image of glomerular VEGF‐A expression in a control cat glomerulus, scale bar 100 μm and (C) VEGF‐A‐corrected total glomerular staining intensity was increased in control compared to diabetic cats. *P = 0.011.

Glomerular VEGF‐A expression was determined in eight diabetic cats and five control cats and was significantly increased in diabetic compared to control cats (Fig 5).

## DISCUSSION

Diabetic nephropathy in cats has previously gone unrecognised. We demonstrate for the first time that glomerular ultrastructural changes that characterise DN may indeed develop in cats. However, the influences of age, sex, and concurrent disease are important factors to consider in these findings.

Three studies (Bartlett et al., 2010; Greene et al., 2014; Trevejo et al., 2018) have evaluated DM as a risk factor for developing CKD, one of which included 1230 cats with CKD and 1230 control cats (Greene et al., 2014), and found no relationship between DM and CKD. Indeed, the study suggested a decreased risk of development of CKD in diabetic cats. However, a more recent retrospective study of 561 cats (67 with CKD and 16 with diabetes) suggested an association between CKD and DM in cats (Pérez‐López et al., 2019). However, all these studies are retrospective cohort or case control studies demonstrating associations only. Whilst it is unlikely that diabetic cats progress to end‐stage or even azotaemic kidney disease, this may be because they do not survive for the same number of years as people with diabetes or because they do not develop nephron loss associated with DN. In diabetic people, the peak incidence of overt DN is 10 to 20 years' post‐diagnosis (Gheith et al., 2016) and therefore it seems likely that cats do not survive long enough with diabetes to progress from early stage DN to later stage disease. In rodent models, glomerular ultrastructural changes are seen within a few weeks of developing diabetes (Dower et al., 2017; Siu et al., 2006). However, it must be noted that rodent models used to study DN are selected for their genetic predisposition to developing DN.

Ultrastructural changes in the glomeruli of diabetic cats were present despite renal histopathological scores being similar between control and diabetic cats. This highlights the need for electron microscopy for fully evaluating renal changes in DN.

The GenC fenestrations are transcytoplasmic pores that allow the unimpeded passage of fluids and small solutes. They largely contribute to hydraulic permeability at the glomerular filtration barrier and thus play an important role in regulating GFR. In a mouse model of diabetes and in people with diabetes, increased fenestration width and decreased fenestration density have been demonstrated (Finch et al., 2022). It was postulated that increased fenestration width was a compensatory mechanism to maintain fenestration surface area. Indeed, fenestration surface area was not significantly different in diabetic compared to control mice. However, in diabetic people, the fenestration surface area was significantly decreased compared to control patients. This would suggest that as the disease progresses, the early compensatory mechanism of increasing fenestration width can no longer maintain the fenestration surface area as there is further fenestration loss. In cats in the present study, we found that fenestration width was not significantly increased in diabetic compared to control cats. It is possible that increasing fenestration width is not a feature of DN in cats or, more likely, the small study number precluded a statistically significant finding. Loss of fenestration density and surface area has been shown to be correlated with reduced glomerular hydraulic permeability and GFR (Finch et al., 2022). A further GEnC fenestration change that may influence hydraulic permeability and GFR is the formation of diaphragmed fenestrations in disease that contribute to resistance to flow (Finch et al., 2022; Greene et al., 2014). The formation of diaphragmed fenestrations was not evaluated in the present study. Glomerular filtration rate and hydraulic permeability were not measured in the present study to determine whether the fenestration loss was affecting filtration function. Serum creatinine was available, which provides a surrogate marker of GFR and was not significantly different in diabetic compared to control cats. However, serum creatinine is an insensitive marker of early reduction in GFR (Finch, 2014).

It is possible that glomerular hypertrophy and hyperfiltration could compensate for decreased filtration function at the filtration barrier. We did not determine glomerular volume in cats in the present study. However, in a previous study of diabetic mice with decreased glomerular hydraulic permeability (a measurement of the ultrafiltration coefficient), glomerular volume was not significantly different from control mice, indicating it was changes in the GEnC fenestration surface area rather than glomerular volume that influenced the glomerular filtration capacity (Finch et al., 2022).

The GBM width was significantly increased in diabetic cats compared to control cats, a well‐established finding in both animal models of diabetes and people (Brown et al., 1983; Damnjanovic et al., 2015; Finch et al., 2022; Marshall, 2016). Indeed, increasing GBM width may be one of the earliest changes in DN in people, documented 1.5 to 2.5 years after diagnosis (Østerby, 1972) or even in the pre‐diabetic state (Lai et al., 2004). The change in GBM width results from changes in its composition. Podocytes and GenCs play a role in maintaining and modifying the GBM. Any damage to these cells can result in alteration of the GBM by accelerating the synthesis of extracellular matrix in the GBM, as well as decreasing degradation. This imbalance between synthesis and degradation leads to an altered composition and GBM thickening (Marshall, 2016). The altered composition will disrupt protein exclusion and therefore contributes to increased protein filtration at the glomerular filtration barrier (Suh & Miner, 2013). All diabetic cats included in the present study were proteinuric. Previous studies have also reported between 38% (Paepe et al., 2015) and 75% (Al‐Ghazlat et al., 2011) of diabetic cats to be proteinuric. Whilst GBM change may have contributed to the development of proteinuria, podocyte injury and dysregulation can also play a key role in aberrant protein exclusion and filtration at the glomerular filtration barrier (Marshall, 2016). Podocyte change including foot process and slit widening are features of DN in people and animal models (Lin & Susztak, 2016; Maezawa et al., 2015; Pagtalunan et al., 1997; Siu et al., 2006; Weil et al., 2011). Podocyte slit and foot process width was higher in diabetic cats although this did not achieve statistical significance, which was likely influenced by the small number of cats included in the study. Proteinuria is a predictor for progressive CKD in cats and whilst it is not generally recognised that cats with diabetes develop progressive CKD, proteinuria itself is a predictor for all‐cause mortality in cats with CKD (Syme et al., 2006). Therefore, monitoring and treating proteinuria in cats with diabetes should be considered an essential part of clinical management.

Vascular endothelial growth factor‐A plays a critical role in GEnC fenestration regulation (Eremina et al., 2003). VEGF‐A exists as different isoforms denoted by the number of amino acids, with VEGF‐121 being the predominant isoform in human glomeruli and VEGF‐164 in mouse glomeruli (Turner et al., 2016). The predominant isoform in the glomeruli of cats is unknown. Studies of VEGF‐A/VEGFR2 pathway manipulations have highlighted that specifically podocyte‐derived VEGF‐A is required for a healthy glomerular endothelial phenotype (Siddiqi & Advani, 2013). Podocyte‐specific overexpression and knockdown of VEGF‐A in mouse models would suggest that it not only maintains fenestrations but also plays a role in contributing to GEnC injury (Kuppe et al., 2018). In the present study, diabetic cats had significantly increased glomerular VEGF‐A expression compared to control cats. In people, glomerular VEGF‐A expression is increased in early stage DN (Dae Ryong et al., 2000) but decreases in late‐stage disease (Baelde et al., 2004). The findings of the present study may suggest that cats have changes similar to early stage DN in people. Tubular epithelial cells can also produce VEGF‐A, with VEGF‐A overexpression being shown to increase serum VEGF‐A concentrations (Kuppe et al., 2018). This mechanism has been proposed to expose GEnCs to higher VEGF‐A concentrations *via* the systemic circulation, which may also influence fenestration density. VEGF‐A is important in both neovascularization and vascular permeability; however, the downstream signalling pathways for these appear to be different. Rac, a GTP‐binding protein, is key in VEGF‐A downstream signalling pathways to induce fenestrations (Eriksson et al., 2003), although this remains to be studied in the glomerulus. Current evidence would suggest that VEGF‐A regulates fenestrations through promoting actin cytoskeleton remodelling and cell membrane lipid rafts (Hunt et al., 2019). In addition to different VEGF‐A isoforms, angiogenic (a) and antiangiogenic (b) splice variants of VEGF165 are also reported, with glomerular overexpression of the antiangiogenic isoform, VEGF165b, decreasing GEnC fenestration density and width (Qiu et al., 2010). This suggests that an intricate balance of both angiogenic and antiangiogenic VEGF‐A is likely to be required to maintain GEnC fenestrae. Therefore, VEGF‐A modulation as a therapeutic option may be challenging and unpredictable. To date, the use of anti‐VEGF antibodies in diabetes has demonstrated the importance of maintaining a normal level of VEGF in the kidneys, as clinical studies have shown that complete elimination of VEGF can be detrimental and accelerate endothelial injury (Tao et al., 2021).

The study has several limitations. Firstly, the small numbers of cats precluded evaluation of associations between the glomerular ultrastructural changes and markers of filtration function such as serum creatinine. Furthermore, the age of the diabetic cats was significantly higher than that of control cats. Fenestration loss in liver sinusoidal endothelial cells is associated with aging and is a proposed mechanism for dyslipidemia in elderly people (Hilmer et al., 2005). It is therefore possible that there is an age‐related decline in GEnC fenestrations, although this has never been studied. Sex differences in GenC fenestrations have not been studied; however, it is possible that this may play a role. Furthermore, it is possible that glomerular VEGF‐A expression may differ between males and females (Baserga et al., 2009). All cats (both diabetic and controls) had concurrent disease, the effect of which on the glomerular endothelium is unknown. Some of the samples had been in storage for a length of time. Most of the samples in the electron microscopy analysis were from the past 5 to 10 years; however, one control sample was collected approximately 20 years prior to evaluation. All of the four additional sections from diabetic cats on which immunohistochemistry was performed to determine glomerular VEGF‐A expression were collected >20 years prior to evaluation. Once fixed, it would not be expected for the samples to deteriorate if stored in the correct fixatives (Shami et al., 2024); however, it is possible that there may be architectural (Likhithaswamy et al., 2022) including ultrastructural (Shami et al., 2024) changes in formalin‐fixed tissue stored for very prolonged periods. Prolonged fixation or storage of paraffin‐embedded sections may decrease immunoreactivity, which may influence immunohistochemistry results (Wester et al., 2000). This is dependent on the epitope, and no specific information is available related to storage effects on VEGF‐A immunoreactivity. Nevertheless, storage effects can be overcome by optimising the antigen retrieval protocol (Wester et al., 2000), and therefore, it was considered unlikely that storage time influenced the results of the VEGF‐A immunohistochemistry staining. When performing TEM to evaluate glomerular ultrastructural changes, glutaraldehyde in cacodylate buffer is considered to be the optimal fixative. Unfortunately, only formalin‐fixed tissue was available in the University of Bristol pathology archives. Any effect that the suboptimal fixative solution had in the study was standardised as all samples evaluated were formalin fixed. We attempted to increase the number of cats we were able to include in the study by deparaffinising previously paraffin‐embedded kidney tissue sections; however, this badly damaged the glomerular architecture and the samples could not be analysed. Considering the limitations, we cannot rule out the effect of these confounding factors on the study findings, and a larger study to evaluate the ultrastructural changes in the glomeruli of cats with diabetes is warranted.

In conclusion, the results of this study provide evidence that diabetic cats may develop glomerular ultrastructural changes, as well as increased VEGF‐A expression, findings that could be consistent with DN in other species. This may contribute to proteinuria, and therefore, UPC should form an important part of diabetic monitoring. The ultrastructural changes may also contribute to a reduction in filtration at the glomerular filtration barrier; however, this may not progress to azotaemia during the lifespan of a diabetic cat even when the disease has been diagnosed for 2 years. Further research is required to confirm this association.

### Author contributions

**H. Reyes‐Hughes:** Investigation; funding acquisition; writing – original draft; methodology; validation; visualization; writing – review and editing; formal analysis; data curation. **E. Bird:** Investigation; funding acquisition; methodology; validation; writing – review and editing; formal analysis; data curation. **C. Neal:** Investigation; writing – review and editing; visualization; software; resources. **R. Noiva:** Investigation; writing – review and editing; methodology; formal analysis. **R. R. Foster:** Methodology; writing – review and editing; supervision; investigation. **S. Satchell:** Investigation; writing – review and editing; methodology; supervision. **N. Finch:** Conceptualization; investigation; funding acquisition; writing – original draft; methodology; validation; visualization; writing – review and editing; formal analysis; project administration; data curation; supervision.

### Funding statement

The authors received financial support from a BSAVA PetSavers student research grant for transmission electron microscopy analysis of kidney samples and BBSRC summer studentship funding for the VEGF‐A immunohistochemistry analysis.

### Conflict of interest

The authors declared no potential conflicts of interest with respect to the research, authorship and/or publication of this article.

## Supporting information


**Supplementary Material 1.** Histological scoring system for kidney tissue

## Data Availability

The data from this study are available in anonymised format only from the corresponding author upon reasonable request.
