# Supplementary material for: Glomerular ultrastructural change and vascular endothelial growth factor‐A expression in diabetic cats
Source: J Small Anim Pract. 2025 Jun 4;66(9):600–8. doi: 10.1111/jsap.13884 (PMC12417099; doi:10.1111/jsap.13884)
Supplement: Supplementary file 1 — Supplementary Material 1. Histological scoring system for kidney tissue [file JSAP-66-600-s001.docx]

**The development of diabetic nephropathy in cats**

**Supplementary material**

Supplementary material 1: Histological scoring system for kidney tissue

| Variable | Scoring evaluation | Score assigned |
| --- | --- | --- |
| % normal parenchyma | <25% | 1 |
|  | 25-50% | 2 |
|  | 51-75% | 3 |
|  | >75% | 4 |
| Tubule degeneration | No tubular degeneration | 0 |
|  | Focal scattered tubular degeneration | 1 |
|  | Multifocal to coalescing degeneration | 2 |
|  | Entire nephron units affected | 3 |
| Interstitial inflammation | No inflammation | 0 |
|  | <25% of the interstitium affected | 1 |
|  | 25-50% of the interstitium affected | 2 |
|  | 51-75% of the interstitium affected | 3 |
|  | >75% of the interstitium affected | 4 |
| % cortical scarring | none | 0 |
|  | <25% | 1 |
|  | 25-50% | 2 |
|  | 51-75% | 3 |
|  | >75% | 4 |
| % medullary scarring | none | 0 |
|  | <25% | 1 |
|  | 25-50% | 2 |
|  | 51-75% | 3 |
|  | >75% | 4 |
